# Assessing the efficiency of danacid as corrosion inhibitor for aluminium in HCl medium: Experimental, theoretical and optimization studies

**DOI:** 10.1016/j.heliyon.2024.e40994

**Published:** 2024-12-06

**Authors:** O.D. Onukwuli, I.A. Nnanwube, F.O. Ochili, J.I. Obibuenyi

**Affiliations:** a Department of Chemical Engineering, Nnamdi Azikiwe University, P.M.B. 5025, Awka, Anambra State, Nigeria; b Department of Chemical Engineering, Madonna University, Akpugo Campus, 402105, Akpugo, Enugu State, Nigeria

**Keywords:** Danacid, RSM, DFT, Activation energy, Inhibition efficiency, Potentiodynamic polarization

## Abstract

The present study examined the corrosion protection of aluminium in 1M HCl by deploying expired danacid, with techniques such as gravimetric, electrochemical, and density functional theory (DFT). Inhibitor characterization was executed with Fourier transform infrared (FTIR) spectroscopy and gas chromatography mass spectrometry (GC-MS), which was supplemented by optimization of parameters with response surface methodology. The results of gravimetric study indicates that the inhibition efficiency (IE) rose with rise in danacid concentration and reduced with rise in temperature. FTIR results shows the presence of heteroatoms in the inhibitor while further analysis by GC-MS showed the presence of heteroatom-carrying compounds such as menthyl acetate, eicosyl vinyl ester, carbonic acid, dotriacontane, n-hexadecanoic acid, among others. The activation energy value obtained from this work suggest that the inhibitor molecule's adsorption on the surface of aluminium follows physical adsorption mechanism. Potentiodynamic polarization (PDP) results portrays the inhibitor as a mixed-type while the results of electrochemical impedance spectroscopy (EIS) tests gave an inhibition efficiency of 95.05 %. RSM results show optimum inhibition efficiency of 93.57 % while artificial neural network (ANN) gave improved predicted values. The DFT results further show that the danacid molecules could effectively inhibit alumina corrosion in aggressive media. Hence, danacid proved to be a viable inhibitor for aluminium corrosion.

## Graphical abstract

**Figure:**
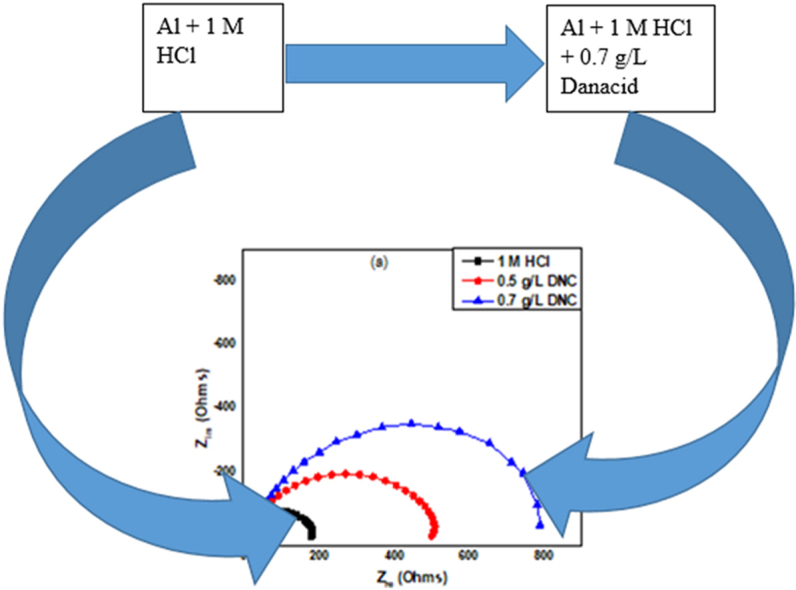

## 1 Introduction

Aluminium (Al) does not occur naturally, it is rather found with other elements and third commonest element that exists, next to silicon (Si) and oxygen (O) and occupies about 7.3 % of the earth's crust [1,2]. It exists as fluoride, oxides, double silicates, and basic sulphates. For the most of its existence, Al and related compounds have as well been the main building material used by the automotive and aviation sectors owing to their high thermal and electrical conductivity, good appearance, low density, cost-effectiveness and light weight. Other usage of aluminium includes lithographic plates, domestic and office furniture, road barriers and signs, sporting goods, machined components, high pressure gas cylinders and ladders and access equipment. Usefulness of aluminium is hindered by corrosion. It corrodes by reacting with the environment [[3], [4], [5]].

Corrosion is the process by which materials deteriorate because of reactions with their environment. It is often seen as the degradation or irreversible destruction of the surface of metal due to chemical reactions involved in the translation of pure metal to a more chemically unchanging form (such as hydroxides, oxides, sulphides) in a corrosion-prone environment. Corrosion-prone setting may be of solid, liquid or gas form. Corrosion of metals is quite a complex and a worldwide phenomenon [[6], [7], [8], [9]]. The settings are called electrolytes, while transference of ions (anions and cations) forms two reactions. In a situation of two different metals in an electrolyte, the less noble metals perform as anode and become corroded while the more noble metal perform as cathode and become protected. In a conducting solution, zinc tends to corrode while copper is more likely to remain protected. This is due to the electrochemical series; zinc has a lower electrode potential, making it more susceptible to oxidation and corrosion, while copper has a higher electrode potential and is less prone to corrosion [10,11].

The high tendency of aluminium and its alloys to resist corrosion is due to the development of a compact, adherent inert oxide film, which is amphiprotic and dissolves to a great extent on exposure of the metal to alkaline or acids solutions. The deterioration of aluminium and its compounds in aqueous solutions comes with substantial cost implication. It is therefore essential to introduce inhibitors to shield the metal from corrosion. Many organic compounds are deployed as corrosion inhibitors for aluminium and its compounds in alkaline and acidic media. The inhibitive performance of these compounds hinge on the chemical composition of the inhibitor, the metal's surface charge, and the type of contact between the molecules of the inhibitor and the surface of the metal. Often, inhibitors perform by sticking to the surface of the metal and creating a coating that shields it. Typically, inhibitors are dispersed from a solution; a portion are part of the preparation of protective coatings [[12], [13], [14], [15], [16]].

Majority of the organic materials deployed as inhibitors are very costly and naturally toxic. Hence, there is need to find non-toxic, eco-friendly, natural and low-cost inhibitors for shielding of alloys and metals from corrosion in aqueous solutions. A viable substitute to these organic compounds has been found to be expired drugs, since they possess the above desirable properties, and have been found to adsorb on metallic surfaces. They create layers and precipitates on the surface of the metal, leading to the obstruction of anodic and cathodic sites. Some of the drugs that have been successfully applied include atenolol drug [17], cimetidine [11], antithyroid drugs [18], among others.

The current study intends to advance the usage of drugs as corrosion inhibitor, by using danacid for such purpose. Danacid tablet is a compound of magnesium trisilicate which is used for the management of hyperacidity, heartburn, dyspepsia, peptic ulcer disease and reflux esophagitis. Due to its adsorptive and eco-friendly properties, expired danacid has been found as a good candidate for corrosion inhibition. In our previous publication, danacid was applied as a corrosion inhibitor of aluminium in sulphuric acid medium [19]. In the present study, it is deployed as a corrosion inhibitor of aluminium in HCl media with the aid of electrochemical impedance spectrometry (EIS), potentiodynamic polarization (PDP), quantum chemical computations, as well as modeling and optimization using artificial neural network (ANN) response surface methodology (RSM).

## 2 Experimental

### 2.1 Preparation of the inhibitor

In this work, varied concentrations of the inhibitor were prepared. Ten grams of the expired drug was mixed with 1 L of HCl solution. The solutions of the inhibitor were set at concentrations of 0.1–0.9 g/L from the stock solution [[19], [20], [21]].

### 2.2 Metals preparation

Aluminum metal of dimension (3 cm × 3 cm) was cut into coupons. The aluminium has the composition: Mg (0.03 %), Al (99.3 %), Fe (0.02 %), Cu (0.03 %), Zn (0.07 %), V (0.04 %), Ti (0.12 %), Si (0.25 %), Mn (0.14 %). The metal preparation procedure had been reported [19,22].

### 2.3 Characterization of the inhibitor

Chemical analysis of the expired danacid was done with a GC-MS (7890A and 5977B MSD model from Agilent Technologies) as well as FTIR spectroscopy (Cary 630 model from Agilent Technologies), as previously reported [10,19,23].

### 2.4 Thermometry

The thermometry of the process had been reported [19,20]. The reaction number (RN) and the inhibitor efficiency (IE) were respectively obtained with Equations (1), (2)) [11,22].

(1) $$RN=\frac{T_{m}-T_{i}}{t}$$

(2) $$IE\left(\%\right)=\left(1-\frac{{RN}_{inh}}{{RN}_{unih}}\right)*100$$

### 2.5 Gravimetric method

The gravimetric method had previously been reported. The corrosion rate (CR), weight loss (Δw), surface coverage, and IE, were respectively computed through the application of Equations (3), (4), (5), (6) as previously reported [[19], [20], [21],[24], [25], [26], [27], [28], [29]].

(3) $$CR=\frac{w_{i}-w_{f}\;}{At}$$

(4) $$\Delta w=w_{i}-w_{f}$$

(5) $$\theta =\frac{\omega_{0}-\omega_{1}}{\omega_{0}}$$

(6) $$IE\%=\frac{\omega_{0}-\omega_{1}}{\omega_{0}}*100$$

1 and 0 correspondingly represent the weight loss values in the danacid-HCl and only HCl medium, w_f_ and w_i_ represent the final and initial weights of the metal. A represents the entire specimen area (cm^2^), and t represents immersion time (h). θ designates the degree of surface coverage.

### 2.6 Estimation of heat of adsorption (Q_ads_) and activation energy (E_a_)

The linearized form of Arrhenius model was deployed to estimate the activation energy of the inhibition process as shown by Equation (7).

(7) Ln(CR)=LnA−(EaR)1T

where CR, E_a_, A, T and R respectively denote the corrosion rate, activation energy, frequency factor, temperature and gas constant. By denoting the metal's corrosion rates at T_2_ and T_1_ as CR_2_ and CR_1_, Equation (8) is obtained [10,24,30].

(8) Ln(CR2CR1)=LnA−(Ea2.303R)(1T1−1T2)

As previously reported [20], Q_ads_ (kJmol^−1^) was calculated with Equation (9).

(9) $$Q_{ads\;}=2.303R\left[\log\frac{\theta_{2}}{1-\theta_{2}}-\log\frac{\theta_{1}}{1-\theta_{1}}\right]*\frac{T_{2}{.T}_{1}}{T_{2}-T_{1}}$$

where R denote the gas constant, θ_2_ and θ_1_ and correspondingly designate the degree of surface coverage at T_2_ and T_1_.

#### 2.6.1 Adsorption isotherms

The θ data was deployed to evaluate the usability of different isotherm models, such as the Langmuir, Flory-Huggins, Frumkin, and Temkin model as respectively depicted by Equations (10), (11), (12), (13), as previously reported [19].

(10) $$\frac{C}{\theta }=\frac{1}{K_{ads}}+C$$

(11) $$\log\left[\left(\frac{\theta }{C}\right)\right]=\log\;K_{ads}+x\;\log\left(1-\theta \right)$$

(12) $$\log\;\left[\left(C\right)\times \left(\frac{\theta }{1-\theta }\right)\right]=2.303\;\log\;K_{ads}+2\alpha \theta$$

(13) $$\theta =-\frac{2.303\;\log\;K_{ads}}{2a}-\frac{2.303\;\log\;C}{2a}$$

where C, θ, $K_{ads}$, x, α respectively denote the concentration of the inhibitor, degree of surface coverage, adsorption equilibrium constant, size parameter and the lateral interaction term. The free energy of adsorption ($\Delta G_{abs}$) was estimated with Equation (14).

(14) $$\Delta G_{abs}=-2.303\;\text{RT}\log\left(55.5K\right)$$

### 2.7 Electrochemical studies

PDP and EIS, three electrochemical techniques, were used in this study, as previously applied by Omotioma et al. [17]. Temperature was maintained at 30 ± 1 °C [19].

### 2.8 Quantum chemical study

Molecular modeling and quantum chemical techniques were used to determine the molecular composition and adhesive properties of the inhibitor (danacid) as previously reported [19,[31], [32], [33]].

### 2.9 Weight loss determination with RSM

RSM was deployed in the experiment design of the weight loss procedure. Inhibitor concentration (IC), Temperature, and time, were the variables used in the design while the response was the inhibition efficiency [19].

### 2.10 ANN and RSM model comparison

Comparison of ANN and RSM was done to evaluate their analytical and valuation capabilities with statistical tools namely, root mean square error (RMSE), standard error of prediction (SEP), mean absolute error (MAE), as shown in Equations (15), (16), (17)) [19].

(15) $$RMSE={\left(\frac{1}{n}\sum_{i-1}^{n}{\left(Y_{pred.,i}-Y_{\exp.,i}\right)}^{2}\right)}^{1/2}$$

(16) $$SEP=\frac{RMSE}{Y_{\exp.\;ave}}*100$$

(17) $$\text{MAE}=\frac{1}{n}\sum_{i=1}^{n}\left|\left(Y_{\exp.,i}-Y_{pred.,i}\right)\right|$$

## 3 Results and discussion

### 3.1 Functional groups of danacid by FTIR

The functional groups found in danacid had previously been reported [19].

### 3.2 Thermometry

Table 1 depicts the effect of the concentration of the expired danacid on the RN and IE. The RN was determined by the fraction of change in temperature to the maximum time attained. The expired danacid's concentration range stretched from 0.1 to 0.9 g/L. The RN decreased with increase in inhibitor concentration (IC). The IE was evaluated as a function of reaction number (in the presence and absence of the inhibitors). The IE increased with increase in IC and reduction in RN [19].

**Table 1:** Influence of danacid concentration on the IE and RN.

IC (g/L)	RN (^o^C/min)	IE (%)
0.0	0.3409	
0.1	0.1052	69.14
0.3	0.0703	79.38
0.5	0.0414	87.86
0.7	0.0156	95.41
0.9	0.0231	93.22

### 3.3 Result of weight loss method

The loss in weight of a metal sample in its area multiplied by the duration the experimental work was carried out defines the rate of metal dissolution. The main merit of this method is that it is convenient and simple to determine corrosion conditions and little inhibitor dosage is needed for additional experiments. The disparities of protection efficiency and dissolution rate in the protected and unprotected media are presented in Table 2. Results displayed in Table 2 shows that danacid is a possible candidate for aluminium protection in acidic environments indicating a slowdown in reaction rate in the inhibited solution in comparison to the uninhibited solution. Close inspection of Table 2 indicates that dissolution rates increased as the temperature was made to rise with the highest values obtained at 323 K in all the systems studied. The corrosion IE rises by increasing the danacids's concentration and is further evident as a result of the large part of active constituents of the inhibitor on the corroding surface of the metal. Conversely, protection efficiency reduced to a great extent as the temperature was increased. This is due to the fact that rise in temperature scatters the extract molecules from the aluminium surface (breaks the heterocyclic bonds found in the danacid, hence, decreasing the surface coverage) [10,25,26].

**Table 2:** Results of weight loss of Al in HCl.

Time (h)	Temperature (K)	Inhibitor conc. (g/L)	Weight loss (g)	CR (g/cm^2^ h)	IE (%)	SC (θ)
5	303	0.0	0.097	0.0022		
		0.3	0.050	0.0011	48.45	0.4845
		0.7	0.036	0.0008	62.89	0.6289
		0.9	0.021	0.0005	78.35	0.7835
	313	0.0	0.109	0.0024		
		0.3	0.059	0.0013	45.87	0.4587
		0.7	0.042	0.0009	61.47	0.6147
		0.9	0.035	0.0008	67.89	0.6789
	323	0.0	0.129	0.0029		
		0.3	0.069	0.0015	46.51	0.4651
		0.7	0.051	0.0011	60.47	0.6047
		0.9	0.045	0.0010	65.12	0.6512
4	303	0.0	0.091	0.0025		
		0.3	0.049	0.0014	46.15	0.4615
		0.7	0.036	0.0010	60.44	0.6044
		0.9	0.028	0.0008	69.23	0.6923
	313	0.0	0.100	0.0028		
		0.3	0.056	0.0016	44.00	0.4400
		0.7	0.041	0.0011	59.00	0.5900
		0.9	0.035	0.0010	65.00	0.6500
	323	0.0	0.114	0.0032		
		0.3	0.065	0.0018	42.98	0.4298
		0.7	0.054	0.0015	52.63	0.5263
		0.9	0.046	0.0013	59.65	0.5965
3	303	0.0	0.069	0.0026		
		0.3	0.041	0.0015	40.58	0.4058
		0.7	0.034	0.0013	50.72	0.5072
		0.9	0.023	0.0009	66.67	0.6667
	313	0.0	0.080	0.0030		
		0.3	0.049	0.0018	38.75	0.3875
		0.7	0.039	0.0014	51.25	0.5125
		0.9	0.033	0.0012	58.75	0.5875
	323	0.0	0.085	0.0031		
		0.3	0.053	0.0020	37.65	0.3765
		0.7	0.044	0.0016	48.24	0.4824
		0.9	0.036	0.0013	57.65	0.5765

### 3.4 Heat of adsorption (Q_ads_) and activation energy (E_a_)

The Q_ads_ and E_a_ for the corrosion control of Al in HCl solution with danacid are shown in Table 3, Table 4. The E_a_ was computed using the Arrhenius model. The E_a_ attained in this study is > 80 kJ/mol, which indicates that the inhibitor molecules’ adsorption on the surface of the metal conforms to the physical mechanism of adsorption [34]. Heat of adsorption is an important thermodynamic property since it shows the straight connection with the degree of surface coverage. Negative values were recorded for the Q_ads_ in this work as shown in Table 4. This shows that the adsorption of the inhibitor on the surface of the metal is exothermic.

**Table 3:** E_a_ for the corrosion control process.

Temperature (K)	CR (mg/cm^2^ h)	E_a_ (kJ/mol)
303	0.889	32.81
313	0.444	
323	1.306	
333	2.083	
343	2.806

**Table 4:** Q_ads_ for the corrosion control process.

IC (g/L)	Q_ads_ (kJ/mol)
0.1	−85.822
0.3	−102.246
0.5	−105.096
0.7	−98.062
0.9	−108.677

### 3.5 Adsorption parameters

**Fig. 1:**
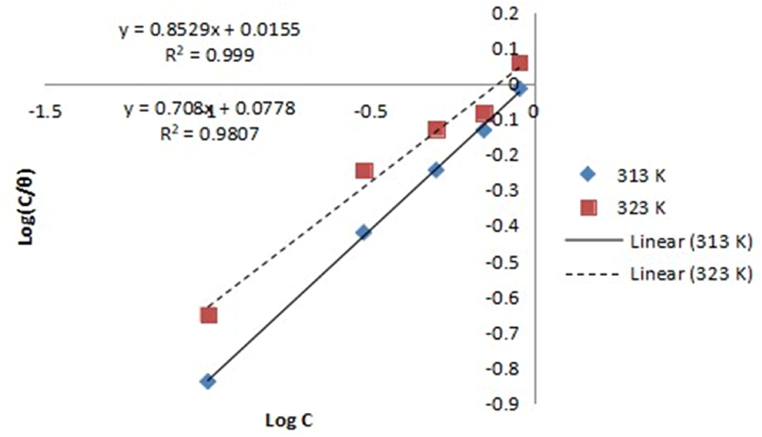
Langmuir model graph.

**Fig. 2:**
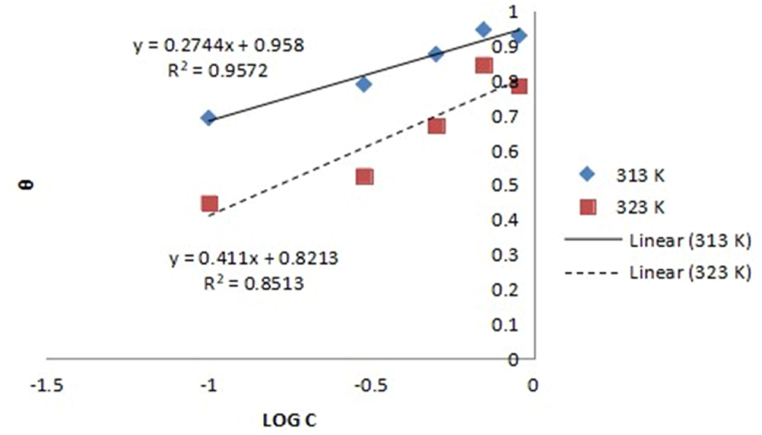
Temkin model graph.

**Fig. 3:**
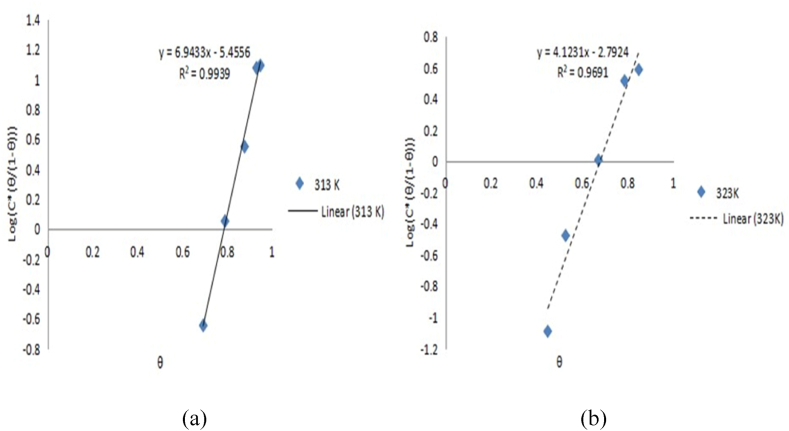
Frumkin model graphs: (a) 313K, (b) 323K.

**Fig. 4:**
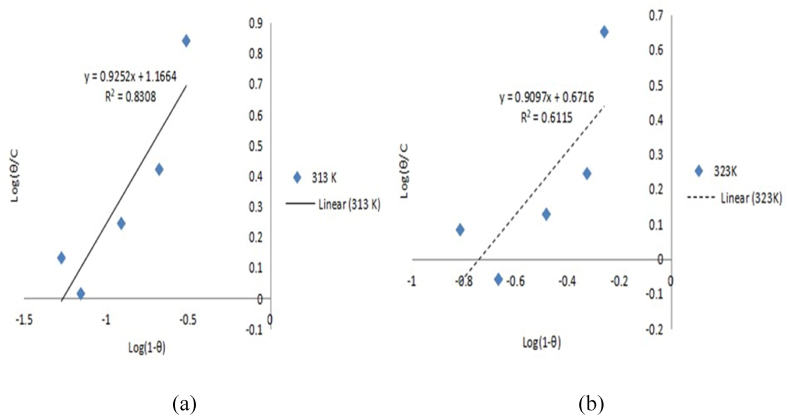
Flory-Huggins model graphs: (a) 313K, (b) 323K.

Langmuir, Frumkin, Temkin, and Flory-Huggins models were used to examine the experimental results for controlling aluminium corrosion in HCl media with expired danacid as inhibitor as presented in Table 5. The Langmuir, Temkin, Frumkin, and Flory-Huggins plots are correspondingly depicted in Fig. 1, Fig. 2, Fig. 3, and Fig. 4(a, b).

**Table 5:** Adsorption parameters.

Adsorption Isotherm	Tempe-rature (K)	R^2^	K_ads_	ΔG_ads_ (kJ/mol)	Isotherm property	
Langmuir Isotherm	313	0.999	0.9649	−10.360		
	323	0.9807	0.360	−8.043		
Temkin Isotherm	313	0.9572	9606008.00	−52.300	a	−8.3929
	323	0.8513	9921.7702	−35.504		−5.6034
Frumkin Isotherm	313	0.9939	0.0043	3.729	α	3.4717
	323	0.9691	0.0613	−3.288		2.0616
Flory-Huggins Isotherm	313	0.8308	14.6690	−17.443	x	0.9252
	323	0.6115	4.6946	−14.941		0.9097

Correlation coefficient values of 0.999 and 0.999 respectively recorded at 313 K and 323 K show that Langmuir model term gave the finest fit to the results of the experiment. By comparison of equations (10), (11), (12), (13)) with the isotherm plots, the adsorption parameters K, a, x, and α were evaluated [10,23]. The isotherms and their corresponding parameter values are displayed in Table 5.

### 3.6 Polarization measurements

**Fig. 5:**
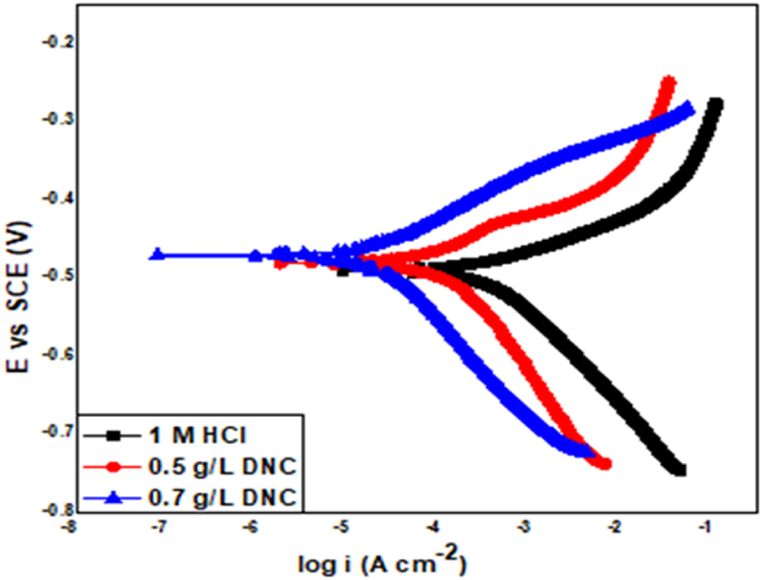
PDP curves of Al in 1 M HCl in the uninhibited and inhibited solution.

PDP measurements were carried out in uninhibited and inhibited acid media containing different concentrations of expired danacid to gain further insight about the behaviour of Al in 1 M HCl. From Fig. 5, it is clear that the presence of danacid suppressed the anodic and cathodic reactions. The Tafel polarization factors recorded from the PDP experiments such as corrosion current density (i_corr_), corrosion potential (E_corr_), cathodic (β_c_) and anodic (β_a_) Tafel slopes are all presented in Table 6. No certain trend is seen in the corrosion potential shifts in danacid's presence; hence, the inhibitor can be regarded as mixed-type inhibitor.

**Table 6:** Polarization parameters.

System	E_corr_ (mV)	I_corr_ (μA/cm^2^)	b_a_ (mVdec^−1^)	b_c_ (mVdec^−1^)	sc (θ)	IE (%)
1 M HCl	−478.4	208.7	88.6	46.8		
1 M HCl+ 0.5 g/L DNC	−477.6	27.4	93.6	54.7	0.8687	86.87
1 M HCl + 0.7 g/L DNC	−472.4	12.8	87.6	42.5	0.9387	93.87

As depicted in Table 6, there are no considerable changes in the β_c_ and β_a_ values in the presence of the inhibitor, hence, cathodic and anodic reactions are not affected and the danacid's inhibition action is majorly due to the geometric obstructive effect implying the decrease of the reaction area on the aluminium surface by obstructing the active reaction sites, which does not affect the corrosion reaction mechanism during the inhibition process. Table 6 reveals that the addition of danacid decreased both cathodic and anodic currents and did not reveal any considerable shift in E_corr_, which also prove that expired danacid is a mixed-type inhibitor [10,19].

The inhibition efficiency (η) of expired danacid was computed with Equation (18).

(18) $$\eta =\left[1-\frac{i_{corr}}{i_{corr}^{0}}\right]\times 100$$

where $i_{corr}^{0}$ and i_corr_ respectively denote the corrosion current densities in the absence and presence of danacid.

### 3.7 EIS measurements

**Fig. 6:**
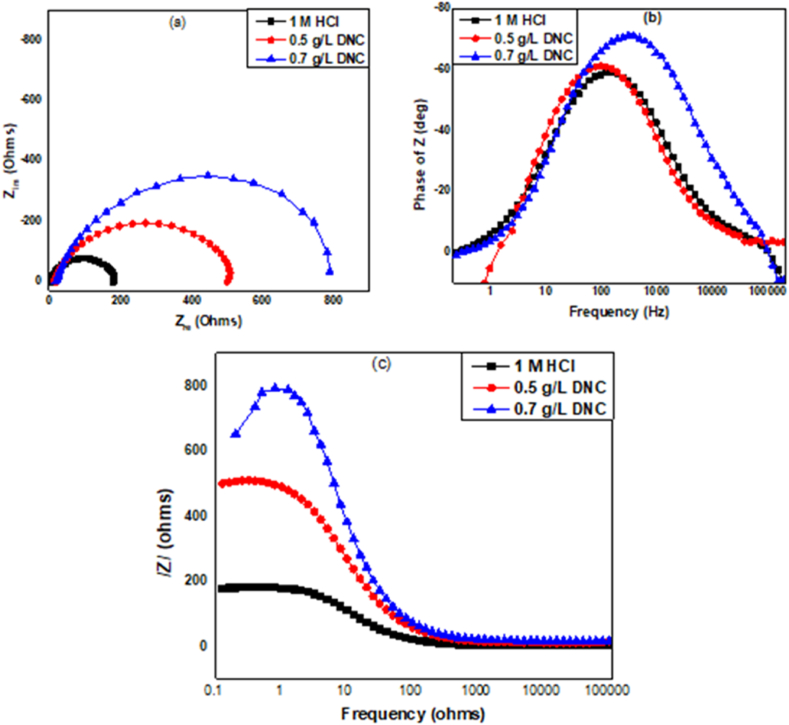
EIS spectra of Al in 1 M HCl in the uninhibited and inhibited media: (a) Nyquist (b) Bode phase angle and (c) Bode modulus plots, respectively.

Measurements of EIS were implemented in 1 M HCl and with different danacid concentrations to give insight into the corrosion behaviour and the adsorption mechanisms. Additionally, EIS was undertaken as rapid and precise technique to evaluate corrosion rates at the aluminium/1 M HCl boundary in the presence and absence of inhibitors. Fig. 6a shows the Nyquist plot while Fig. 6b and c respectively shows the Bode phase angle and Bode modulus plots without and with two concentrations of the inhibitor. Table 7 gives the values of the EIS parameters calculated by fitting the EIS spectra along with the inhibition efficiency (IE, %) values computed with Equation (19).

(19) $$IE\left(\%\right)=\frac{R_{ct}^{i}-R_{ct}^{0}}{R_{ct}^{i}}\times 100$$

where $R_{ct}^{i}$ and $R_{ct}^{0}$ are charge transfer resistances in presence and absence of inhibitor, respectively.

**Table 7:** Impedance parameters of Al in HCl.

System	R_s_ (Ωcm^2^)	R_ct_ (Ωcm^2^)	n	C_dl_ (Fcm^2^)	IE (%)
1 M HCl	1.723	39.7	0.88	7.124E-5	
1 M HCl + 0.5 g/L DNC	1.686	532.4	0.88	7.221E-5	92.54
1 M HCl + 0.7 g/L DNC	1.716	802.7	0.89	7.172E-5	95.05

As presented in Fig. 6, the Nyquist plots demonstrate similarity in behaviour with and without the inhibitor signifying that their existence in 1 M HCl did not change the mechanism of the process. It is worthy of note that the Nyquist semicircles diameter in the inhibited media rise gradually, this became more noticeable upon adding cumulative amounts of danacid to the 1M HCl as demonstrated by a substantial rise in charge transfer resistance values, with associated reductions in C_dl_ values as shown in Table 7. The decrease in the value of C_dl_ in danacid's presence is due to the reduction in local dielectric constant and a rise in the electrical double layer's thickness, owing to the shift taking place between inhibitor and water molecules throughout the adsorption process [30]. From the results displayed in Table 7, the IE increased from 92.54 to 95.05 % as the concentration of danacid was increased from 0.5 to 0.7 g/L.

### 3.8 GC-MS characterization of danacid

GC-MS results of danacid had previously been reported. The peaks show numerous heterocyclic compounds found in danacid. The components found include 1-methyl-4-(1-methyl ethyl)-Cyclohexanol, dl-Menthol, trans-13-Octadecenoic acid, Dotriacontane, 9,12-Octadecadienoic acid, 1-chloro- Hexadecane, n-Hexadecanoic acid, 1-chloro-Octadecane, eicosyl vinyl ester, buty l,2-methylpropyl ester, Carbonic acid, tetradecyl ester, cis-Vaccenic acid, 9-Octadecenoic acid, among others [19].

### 3.9 Theoretical modelling

**Fig. 7:**
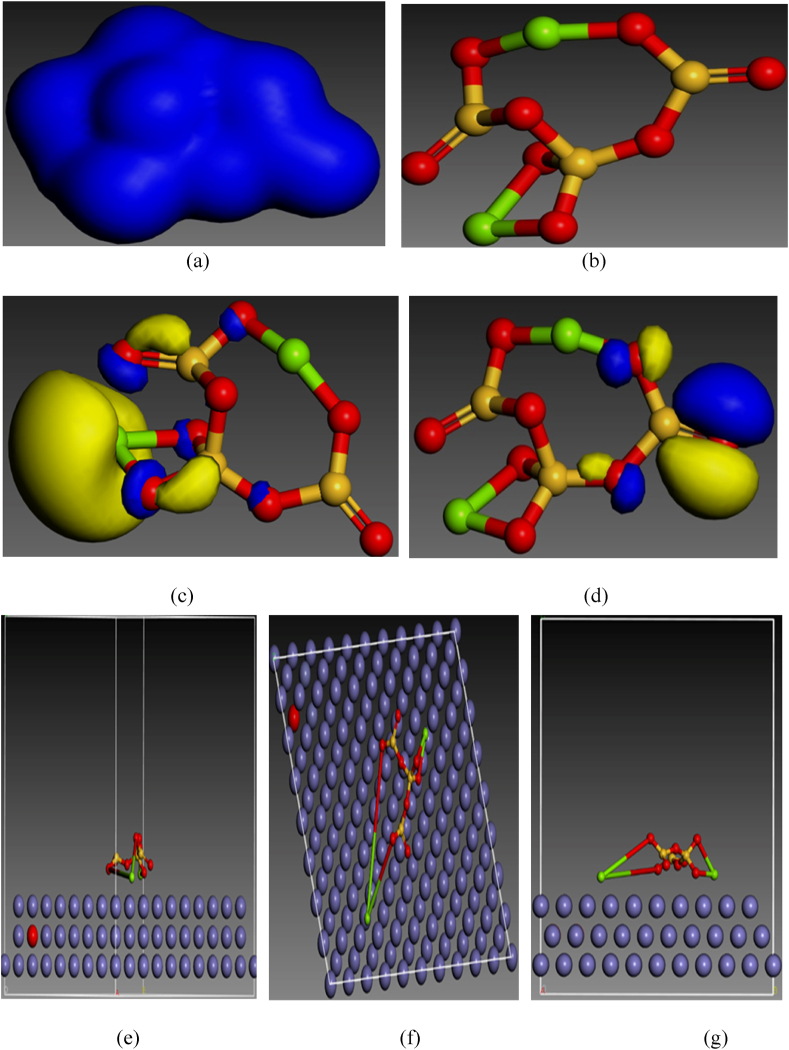
Danacid (magnesium trisilicate (Mg_2_O_8_Si_3_) model: (a) Electron density, (b) Optimized structure, (c) LUMO, (d) HOMO, (e) Side view, (f) Top view, (g) Front view.

The IE of expired danacid is credited to its adsorption on aluminium surface. In order to show the relationship between the inhibiting property and quantum chemical parameters of expired danacid, DFT computations were made with DFT electronic structure programme DMol^3^ executed in Materials Studio Software. The HOMO and LUMO obtained from the optimized molecular structure are depicted in Fig. 7. It has been well established that the reactivity of an inhibitor can be characterized in terms of its HOMO and LUMO. The HOMO depicts the electron donation while LUMO depicts the electron acceptance capability of the inhibitor molecules. From the frontier orbital theory's postulation, E_HOMO_ represents a species' aptitude to donate electrons, signifying that species with higher value of E_HOMO_ are more likely to achieve the best inhibition efficiency. On the other hand, E_LUMO_ depicts a species' ability to accept electrons, hence, an effective inhibitor usually has low values of E_LUMO_. A molecule's energy gap (ΔE) is represented by the difference between E_HOMO_ and E_LUMO_ of the molecule. Low values of ΔE signifies that a molecule is likely to give high inhibition efficiency. The electron density, optimized structure, LUMO, HOMO, side view, top view, and front view of danacid (Mg_2_O_8_Si_3_) molecule on the Al surface are correspondingly shown in Figures (7(a, b, c, d, e, f, g)) [10]. The DFT parameters are depicted in Table 8.

**Table 8:** DFT parameters.

Inhibitor (molecule)	E_HOMO_	E_LUMO_	Energy gap (eV)	Molecular mass (gM^−1^)	Adsorption Energy (eV)
Danacid	−6.026	−3.703	2.323	260.857	−137

To assess the collaboration between the inhibitor molecules and the Al surface, the adsorption energy (E_ads_) of each scheme was computed with Equation (20).

(20) $$E_{\text{Interact}}=E_{\text{total}}\;–\;\left(E_{\text{DNC}}+E_{\text{Al}}\right)$$

E_total_ is considered as the total energy of the area investigated, comprising molecules of danacid and the aluminium surface, E_DNC_, E_Al_ and E_total_ represent an individual molecule's strength on the Al slab.

### 3.10 Results of RSM for Al in HCl media

From Table 9, the maximum inhibitor efficiency for the corrosion protection of Al in HCl was documented as 94.65 %, at the IC of 0.7 g/L, temperature of 313 K, and time of 4 h. The high IE value signifies that the inhibitor is fitting for checkmating corrosion of aluminium in HCl media. There is also an observed rise in the concentration of the inhibitor with rise in IE. It may be related to the nature and effect of molecular construction on their inhibition properties.

**Table 9:** RSM result for corrosion protection of Al in HCl with danacid.

Std	Run	Factor 1 A: IC g/L	Factor 2 B: Temperature K	Factor 3 C: Time h	Response IE %
2	1	0.9	303	3	81.03
1	2	0.5	303	3	68.11
7	3	0.5	323	5	66.14
9	4	0.5	313	4	87.63
12	5	0.7	323	4	84.64
14	6	0.7	313	5	94.23
5	7	0.5	303	5	79.55
10	8	0.9	313	4	92.98
20	9	0.7	313	4	94.65
3	10	0.5	323	3	56.97
11	11	0.7	303	4	88.77
19	12	0.7	313	4	94.65
18	13	0.7	313	4	94.65
17	14	0.7	313	4	94.65
15	15	0.7	313	4	94.65
16	16	0.7	313	4	94.65
13	17	0.7	313	3	91.67
4	18	0.9	323	3	77.01
6	19	0.9	303	5	83.14
8	20	0.9	323	5	79.95

Table 10 displays the ANOVA model of the inhibitor efficiency of Al in HCl. The F-value of 47.59 indicates the model is vital since there is 1 out of 100 probabilities that an F-value of up to 47.59 could ensue owing to noise. P-values <0.0500 specify model components are vital. In this study C^2^, B^2^, A^2^, AC, AB, A, B, C are vital model terms. The predicted R^2^ of 0.8447 is in decent vicinity with the Adjusted R^2^ of 0.9567; i.e. the variance is < 0.2. Adequate Precision ratio of 22.767 designates a satisfactory signal. The model obtained can be deployed to explore the design space [[35], [36], [37], [38], [39], [40], [41], [42], [43]].

**Table 10:** ANOVA of Quadratic model.

Source	Sum of Squares	df	Mean Square	F-value	p-value	
Model	2284.92	9	253.88	47.59	<0.0001	significant
A-Inhibitor concentration	310.36	1	310.36	58.18	<0.0001	
B-Temperature	128.81	1	128.81	24.15	0.0006	
C-Time	79.64	1	79.64	14.93	0.0031	
AB	37.58	1	37.58	7.05	0.0241	
AC	30.26	1	30.26	5.67	0.0385	
BC	0.2592	1	0.2592	0.0486	0.8300	
A^2^	126.09	1	126.09	23.64	0.0007	
B^2^	295.80	1	295.80	55.45	<0.0001	
C^2^	46.82	1	46.82	8.78	0.0142	
Residual	53.35	10	5.33			
Lack of Fit	53.35	5	10.67			
Pure Error	0.0000	5	0.0000			
Cor Total	2338.27	19				
Std. Dev.	2.31		R^2^		0.9772	
Mean	84.99		Adjusted R^2^		0.9567	
C.V. %	2.72		Predicted R^2^		0.8447	
			Adeq Precision		22.7673	

### 3.11 Mathematical models of the IE

The coded mathematical model for this study is given as Equation (21). It is valuable for classifying the comparative impact of the factors by comparison of the factor coefficients. The model in terms of coded factors could be deployed to make estimates about the response for specified levels of each factor [11,18].

(21) $$\text{IE}=+95.62+5.57\;A-3.59\;B+2.82\;C+2.17\;\text{AB}-1.95\;\text{AC}-6.77A^{2}-10.37\;B^{2}-4.13\;C^{2}$$

The equation in terms of actual factors is presented as Equation (22)

(22) $$\begin{array}{c} \text{IE}=-9944.83889-{35.46102\;}^{*}\;\text{Inhibitor}\;\text{concentration}+63.87921^{*}\\ \text{Temperature}+48.27441^{*}\;\text{Time}+1.08375^{*}\;{\text{Inhibitor}\;\text{concentration}}^{*}\;\\ \begin{array}{c} \text{Temperature}-9.72500^{*}\;{\text{Inhibitor}\;\text{concentration}}^{*}\;\text{Time}-{\;169.28409}^{*}\;\\ {\text{Inhibitor}\;\text{concentration}}^{2}-0.103714^{*}\;{\text{Temperature}}^{2}-4.12636^{*}\;{\;\text{Time}}^{2} \end{array} \end{array}$$

### 3.12 Graphical evaluation of the IE

**Fig. 8:**
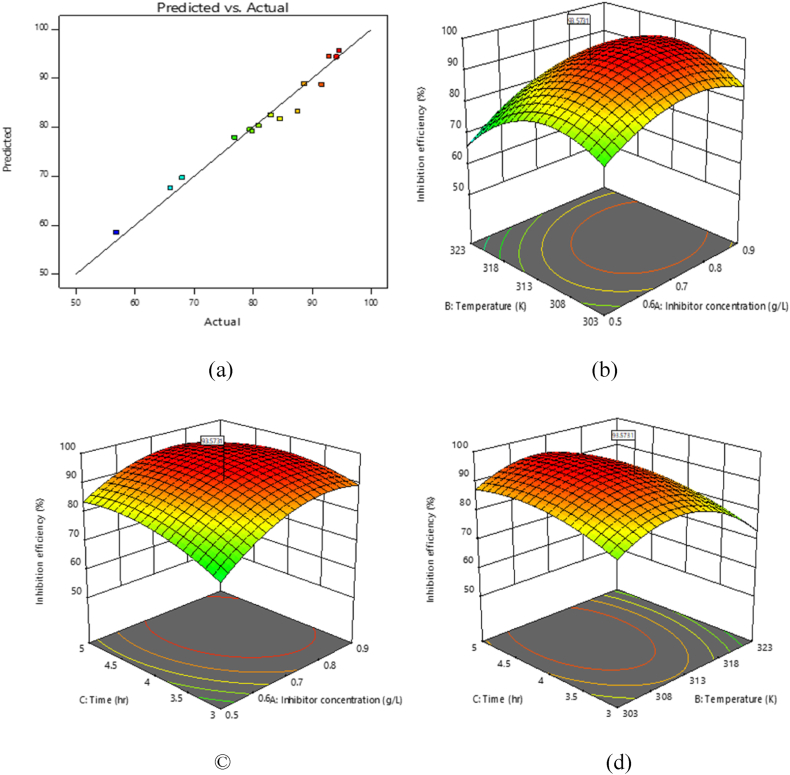
(a) Experimental against predicted values (b) IE against IC and temperature (c) IE against IC and time (d) IE against temperature and time.

Fig. 8(a–d) show the graphical results of IEs of danacid for controlling aluminium corrosion in HCl media. Feasibility of the inhibitor was defined with predicted against actual IE and 3-D graphs. Fig. 8a shows the plot of predicted versus actual IE. The points aligned on the best fitted line, signifying that the model attained successfully defined the experimental IE of danacid [11].

The 3-D graphs reveal the combined effect of temperature, IC and time on the IE of danacid. At the different optimum values of the parameters, the IE of danacid for the corrosion protection of aluminium in HCl medium was recorded as 93.57 %. The high IE value recorded confirms the appropriateness of the inhibitor.

### 3.13 ANN analysis

**Fig. 9:**
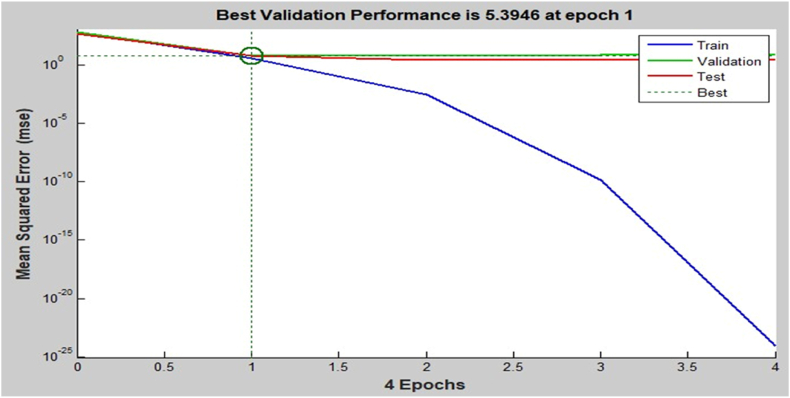
Performance plot.

**Fig. 10:**
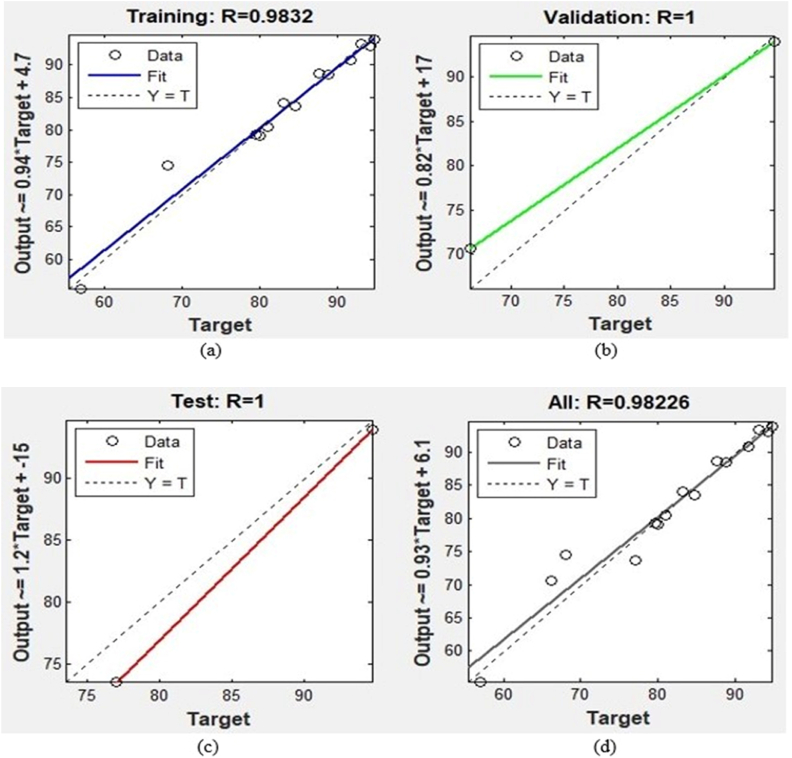
Regression plots showing: (a) training, (b) validation, (c) testing, and (d) overall plot.

The performance graph for controlling Al corrosion in HCl is displayed in Fig. 9, showing 4 epochs with the finest validation value of 5.3946 at the 1st epoch signifying that ANN is fitting for calculating the IE of danacid [11,38]. The regression graphs are shown in Fig. 10(a–d). These show that ANN is an appropriate predictive tool for the inhibition efficiencies obtained.

### 3.14 Evaluation of ANN and RSM models

The evaluation of ANN and RSM results is displayed in Table 11. A comparative analysis was implemented to establish the predictive abilities of ANN and RSM using some statistical models as presented in Table 12. From the statistical investigation performed, ANN gave improved estimate compared to RSM, as authenticated by the lesser values of error parameters such as MAE, RMSE, and SEP [20].

**Table 11:** ANN and RSM predicted results for corrosion protection of Al in HCl.

Std	Run	Factor 1 A: IC g/L	Factor 2 B: Temperature K	Factor 3 C: Time h	Response 1 IE %		
					Experimental	RSM values	ANN values
2	1	0.9	303	3	81.03	80.29	80.8682
1	2	0.5	303	3	68.11	69.59	68.7234
7	3	0.5	323	5	66.14	67.61	66.8716
9	4	0.5	313	4	87.63	83.28	87.0722
12	5	0.7	323	4	84.64	81.66	84.2616
14	6	0.7	313	5	94.23	94.32	93.2762
5	7	0.5	303	5	79.55	79.48	79.477
10	8	0.9	313	4	92.98	94.42	92.1012
20	9	0.7	313	4	94.65	95.62	93.671
3	10	0.5	323	3	56.97	58.44	58.2518
11	11	0.7	303	4	88.77	88.84	88.1438
19	12	0.7	313	4	94.65	95.62	93.671
18	13	0.7	313	4	94.65	95.62	93.671
17	14	0.7	313	4	94.65	95.62	93.671
15	15	0.7	313	4	94.65	95.62	93.671
16	16	0.7	313	4	94.65	95.62	93.671
13	17	0.7	313	3	91.67	88.67	90.8698
4	18	0.9	323	3	77.01	77.80	77.0894
6	19	0.9	303	5	83.14	82.40	82.8516
8	20	0.9	323	5	79.95	79.20	79.853

**Table 12:** Comparison of ANN and RSM models.

Parameters	RSM	ANN
RMSE	1.6330	0.7617
SEP	1.9215	0.8963
MAE	1.2630	0.6698

### 3.15 Optimum parameters and validation

The results depicted in Table 13 shows the optimum concentration of the inhibitor, temperature, time and IE of danacid. Value of optimum IE was obtained as 93.57 %, which indicates that the inhibitor is appropriate for controlling aluminium corrosion in HCl media. The result obtained here was validated with a percentage deviation of 0.68 %.

**Table 13:** Optimum values.

Media	Optimum IC (g/L).	Optimum temperature (K)	Optimum time (h)	Optimum IE (%)
Al in HCl with danacid	0.67	313.36	3.80	93.57

## 4 Conclusion

From the results obtained in this work, expired danacid revealed excellent IE for aluminium in 1 M HCl medium. The IE of danacid increased with increase in its concentration and got to 78.35 % at 0.9 g/L, from the gravimetric study. The IE was however found to reduce with increase in temperature. Polarization results show that the expired danacid acts as a mixed-type inhibitor. The adsorption study carried out demonstrate that the inhibition process followed Langmuir isotherm. PDP measurements indicate rise in charge transfer resistance and IE with rise in IC, reaching an IE of 93.87 % at 0.7 g/L. The value of activation energy recorded in this work indicates that the inhibitor molecule's adsorption on the aluminium surface conforms to the mechanism of physical adsorption. Results of computational calculations using density functional theory show good reactivity of expired danacid on the aluminium surface and correlates with the results obtained by electrochemical measurements. Optimization of process parameters established the optimum points for the inhibition process while ANN modeling improved the inhibition efficiency values predicted by RSM. Hence, danacid proved to be a viable inhibitor for aluminium corrosion.

## CRediT authorship contribution statement

**O.D. Onukwuli:** Visualization, Supervision, Project administration. **I.A. Nnanwube:** Writing – review & editing, Writing – original draft, Software, Formal analysis, Data curation. **F.O. Ochili:** Validation, Resources, Methodology, Investigation, Conceptualization. **J.I. Obibuenyi:** Resources, Investigation.

## Data availability

Data will be made available on request.

## Funding

This research received no funding.

## Declaration of competing interest

The authors declare that they have no known competing financial interests or personal relationships that could have appeared to influence the work reported in this paper.
